# Privacy-Preserving Data Aggregation Protocols for Wireless Sensor Networks: A Survey

**DOI:** 10.3390/s100504577

**Published:** 2010-05-04

**Authors:** Rabindra Bista, Jae-Woo Chang

**Affiliations:** Department of Computer Engineering, Chonbuk National University, Chonju, Chonbuk 561-756, Korea; E-Mail: jwchang@chonbuk.ac.kr

**Keywords:** wireless sensor networks, privacy-preserving data aggregation, eavesdropping, accuracy, energy efficiency

## Abstract

Many wireless sensor network (WSN) applications require privacy-preserving aggregation of sensor data during transmission from the source nodes to the sink node. In this paper, we explore several existing privacy-preserving data aggregation (PPDA) protocols for WSNs in order to provide some insights on their current status. For this, we evaluate the PPDA protocols on the basis of such metrics as communication and computation costs in order to demonstrate their potential for supporting privacy-preserving data aggregation in WSNs. In addition, based on the existing research, we enumerate some important future research directions in the field of privacy-preserving data aggregation for WSNs.

## 1. Introduction

Recently, wireless sensor networks (WSNs) [1–5] have been regarded as not only one of the eight technologies that could save the world [6], along with nuclear waste neutralizers, but also one of the ten emerging technologies that will change the world [7]. A WSN consists of a large number of spatially distributed autonomous resource-constrained tiny sensor devices [8] which are used to cooperatively monitor physical or environmental conditions, such as heat, temperature, sound, vibration, pressure, motion or pollutants, at different locations. WSNs have some unique features, for instance, limited power, ability to withstand harsh environmental conditions, ability to cope with node failures, mobility of nodes, dynamic network topology, communication failures, heterogeneity of nodes, large scale of deployment and unattended operation. Although sensor nodes forming WSNs are resource-constrained, *i.e.*, have limited power supplies, slow processors and less memory, they are widely used in many civilian application areas, including environment and habitat monitoring, healthcare applications, home automation, traffic control and in military applications such as battlefield surveillance.

Because data from sensor nodes are correlated in terms of time and space, transmitting only the required and partially processed data is more meaningful than sending a large amount of raw data. In general, sending raw data wastes energy because duplicated messages are sent to the same node (implosion) and neighboring nodes receive duplicate messages if two nodes share the same observing region (overlap). Thus, data aggregation, which combines data from multiple sensor nodes, has been actively researched in recent years. An extension of this approach is in-network aggregation [9–16] which aggregates data progressively as it is passed through a network. In-network data aggregation can reduce the data packet size, the number of data transmissions and the number of nodes involved in gathering data from a WSN.

Another issue of WSNs is how to preserve sensitive measurements taken from everyday life where data privacy becomes an important aspect. In many scenarios, the confidentiality of transported data can be considered critical. For instance, data from sensors might measure patients’ health information, such as heartbeat and blood pressure details. Also, as mentioned in [17], a future application might measure household details, such as power and water usage, computing average trends and making local recommendations. Since all data are transported wirelessly between sensor nodes, they are typically prone to interception and eavesdropping. Speaking broadly, there are two types of privacy concerns in WSNs: internal privacy and external privacy. The former is about maintaining the data privacy of a sensor node from other trusted participating sensor nodes of the WSN, whereas the latter means that the sensed data is protected from outsiders (adversaries). Data privacy can be simply defined as a process in which private data can be overheard and decrypted by adversaries or other trusted participating sensor nodes, but it can still provide a mechanism that prevents them from recovering sensitive information, *i.e.*, control disclosure of any information about the data. To achieve data privacy, it is required to protect transmission trend of a node’s private data from its neighboring nodes. This is because the neighboring nodes can always overhear the sum of the private data and a fixed unknown number, *i.e.*, an encryption key.

Therefore, in the field of WSNs, privacy-preserving data aggregation is becoming a hot issue in academia. To address this issue, some protocols have been proposed by researchers at various universities and institutions. In this paper, we provide a comprehensive summary and comparison of the existing privacy-preserving data aggregation protocols for WSNs. To the best of our knowledge, only the survey by Li *et al.* [18] is related to privacy-preserving data aggregation protocols. In [18], the authors reviewed privacy-preserving techniques for protecting two types of private information: data-oriented and context-oriented privacy. Our work differs from the existing survey as follows. First, our work focuses only on data privacy in order to provide detailed overviews of the existing privacy-preserving data aggregation protocols for WSNs. Secondly, they did not provide a comprehensive comparison of the protocols in terms of such metrics as types of privacy. Finally, they did not cover the most recent and important protocols in their survey, for example Conti *et al.* [19].

In this paper, we first explain the essential design principles, issues and challenges of privacy-preserving data aggregation protocols for WSNs. Next, we classify them, based on the types of data aggregators as well as the techniques to achieve data privacy. In addition, we make an extensive comparison of the existing privacy-preserving data aggregation protocols for WSNs, in terms of parameters such as communication and computation costs. Finally, we provide some future research directions in the field of privacy-preserving data aggregation for WSNs.

The rest of this paper is organized as follows. In Section 2, we present some major application areas of privacy-preserving data aggregation protocols for WSNs. Section 3 proceeds with the design principles, issues and challenges. In Section 4, we classify and summarize the existing privacy-preserving data aggregation protocols for WSNs. A comparison of the protocols is presented in Section 5. Section 6 lists future research directions on privacy-preserving data aggregation for WSNs. Concluding remarks are in Section 7.

## 2. Application Areas

In this section, we briefly explain some major application areas of privacy-preserving data aggregation (PPDA) protocols where the leakage of sensor data is a critical issue for WSN users. People might not agree to allow an application to intrude on their personal domain if the privacy of the collected information is not protected. Some common application areas of the PPDA protocol are health monitoring, military surveillance and private households.

- *Health Monitoring:* There are two main health monitoring applications for WSNs. First, there is athletic performance monitoring such as tracking a person’s pulse and respiration rate via wearable sensors. Secondly, using health sensors, we can monitor the health of patients, e.g., personal weight, blood sugar level, blood pressure, etc. These sensor measurements of people’s health data should be kept private and hidden from other people during transmission with aggregation to the sink node.
- *Military Surveillance:* In military communications, we can use WSNs to replace guards and sentries around defensive perimeters, keeping soldiers out of harm’s way, to locate and identify targets for potential attacks and to support attacks by locating friendly troops and unmanned vehicles. Therefore, the privacy of the sensor data is always critical and it should be preserved during aggregation.
- *Private Households:* As mentioned in PDA [17], wireless sensors could be placed in houses in order to collect statistics about water, gas and electricity consumption within a large neighborhood. The aggregated population statistics may be useful for individuals, businesses and government agencies for resource planning purposes and usage advice. However, the individual sensor readings could reveal the daily activities of a household, such as when all family members are absent or when someone is taking a shower; *i.e.*, different water appliances have distinct signatures of consumption that can reveal their identity. Hence, we need a way to collect the aggregated sensor readings while preserving data privacy.

## 3. PPDA Protocol Designing Principles, Issues and Challenges

WSNs can be deployed in different private data generating fields, as we mentioned in the previous section. PPDA protocols to design supporting sensitive data aggregation face multiple issues and challenges because of bandwidth limited WSNs with resource-constrained sensor nodes. So, a PPDA protocol designed for WSNs must address the features mentioned below in order to be acceptable in the WSN domain.

- *Eavesdropping and privacy preservation:* Eavesdropping is an attack in which an attacker or a curious individual attempts to obtain private information by overhearing transmissions over its neighboring wireless links. Eavesdropping threatens the privacy of data held by an individual sensor node. In opposition, privacy preservation ensures data privacy against both trusted sensor nodes and adversaries. There are some techniques which can prevent revealing the actual data of a sensor node to other sensor nodes and adversaries even though they overhear the sensor data. For example, the PDA proposed two new trends of data transmission to hide actual sensor data [16]. They are CPDA and SMART protocols. To maintain data privacy, in CPDA, seeds (real numbers) are mixed with actual sensor data before they are sent to a parent node. In SMART, the data of a sensor node is divided into small pieces which are sent to a neighboring node. However, such techniques generate unnecessary data traffics which cause a high cost for resource-constrained sensor nodes.
- *Data pollution and data integrity:* Another type of attack is data pollution, in which an attacker tampers with the intermediate result of sensor data during data aggregation at an aggregator. The purpose of the attack is to make a base station receive a wrong aggregation result, thus resulting in improper or wrong decisions. Because the result of data aggregation is used to make critical decisions, a base station or a user needs to attest to the integrity of the aggregated result before accepting it. Ensuring the correctness of the received aggregated data is highly desirable in civilian applications. Therefore, it is necessary to protect the aggregation results from being polluted by attackers. Moreover, if data pollution can be detected as soon as possible, its impact in the final aggregated result can be avoided. To resolve the problem, a commitment-and-attestation technique is presented in the SDAP and the SIA for secure data aggregation [23,24]. But, the commitment-and-attestation technique delays the decision making process because it needs the searching cost of *O(log N)* to detect the spot of data pollution and ensure data integrity, where *N* is the total number of sensor nodes in a network. In addition, it needs a significant amount of resources for resource-limited sensor nodes.
- *Efficiency:* In WSNs, data aggregation should achieve both bandwidth and energy efficiency through in-network processing. In private data aggregation protocols, an additional communication overhead cannot be avoided when additional features are realized. However, additional overheads due to the resource-constrained nature of sensor nodes, such as communication cost, computation cost, memory and payload size, should be kept to a minimum. The privacy homomorphism technique performs privacy-preserved data aggregation by using the minimal amount of sensor resources [33,35]. However, the privacy homomorphism technique is not flexible enough to include other features, like supporting a variety of aggregation functions and data integrity within a system. Because most existing PPDA protocols [17,19,31] are not seriously concerned about the efficient usage of the resources, it is a challenging task to devise an elastic PPDA protocol for WSNs by considering the resource limitations of sensor nodes.
- *Accuracy:* Because WSNs are not always reliable, it cannot be expected that all sensor nodes will reply to all requests. Because many packets may be dropped during data transmissions through wireless links, the final aggregated result of the sensor data must be properly derived. To provide an accurate sensor data aggregation result to the users, a proper mechanism is required to know which sensor nodes contribute to the aggregated result. For this, Castelluccia *et al.* proposed a technique in which the node identifiers (*IDs*) of contributed sensor nodes are appended to the payloads before sending packets to the sink node [35]. However, due to the limitation of payload size for common sensor nodes like Mica Motes [8], the limited number of *IDs* of sensor nodes can be transmitted after data aggregation. Therefore, the accuracy of the final aggregated result can be affected. As a result, how to compute an accurate aggregated result of sensor data in WSNs is an issue to PPDA protocol designers.
- *QoS support:* Supporting QoS (Quality of Service) is one of the fundamental requirements of most applications [20]. The level of QoS can be varied according to the type of an application. For example, delays are not tolerable while retrieving patients’ health measurements whereas some level of delay is acceptable during the collection of data from private households. There are some features for which an optimal level of QoS can be considered in designing a PPDA protocol for WSNs, such as privacy level, query response time, data-loss resiliency, availability and bandwidth allocation. For example, most of the current PPDA protocols use a cryptographic technique to achieve privacy-preserving data aggregation. Due to the involvement of encryption and decryption processes, data retrieving process is always slow. Therefore, it is a difficult task for protocol designers to define QoS level for different features and include them in the protocol because the limitation of available resources should be considered.
- *Idle time:* The common principle of data aggregation scheme is to create a data aggregation tree for sensor data collection. During data collection, the sensor nodes in the lowest level send their data to upper-level sensor nodes in an active state and they become inactive. The upper-level sensor nodes receive data from the lowest-level sensor nodes which privately aggregate the received data, send them to their parent nodes and become inactive. This process continues until the partially aggregated data from all sub-trees reach to the sink node. On the other hand, because of data privacy concern, two protocols proposed by He *et al.* and Conti *et al.* [17,19] request all the upper-level sensor nodes to be in a listening phase until receiving data from their respective lower-level sensor nodes. It leads to increase in a duty cycle and an idle time for the upper-level sensor nodes. A long duty cycle and a long idle time of sensor nodes per epoch will shorten the lifetime of a WSN. The listening phase nearly consumes as much power as the data reception phase. For example, QUASAR [21] shows a Berkeley mote whose transmission range is set to 20 m. It can provide a transmission rate of 19.2 kbps where the power consumption of data transmission, data reception, and data listening are 14.88 mW, 12.50 mW and 12.36 mW, respectively. Therefore, one of the requirements of protocols is how to manage duty cycling and data listening to a minimum time for the sensor nodes of different levels.
- *Dynamism:* Joining new nodes and leaving the existing nodes are common scenarios in a WSN. The changing conditions of the network must be properly handled in the expense of a minimum configuration cost. For this, it is necessary to use dynamic routing protocol, memory allocation and key distribution in the network while designing a protocol. However, a lot of resources are consumed to operate a dynamic network consisting of sensor nodes with limited power, memory and computational speed.
- *Aggregation functions:* Sensor data can be used to make a critical decision by knowing the patterns and trends of the generated data. Therefore, a protocol should support many aggregation functions, such as *Sum, Average, Count, Standard Deviation, Variance, Min, Max, Median* and *Histogram*. Among them, the *Sum, Average and Count* are easy to compute in privacy-preserving data aggregation. On the other hand, both *Min* and *Max* functions are very difficult tasks because the privacy of data cannot be maintained while comparing the data of two sensor nodes.

In conclusion, the limited resources of sensor nodes are a main challenge for privacy-preserving data aggregation protocols to support the features mentioned above for WSNs.

## 4. Classification of PPDA Protocols

In this section, we present the classification criteria. Based on that, we classify the existing PPDA protocols for WSNs and review them. Work related to security issues in data aggregation [22–24] is out of the scope of this research.

### 4.1. Criteria of classification

In a privacy-preserving data aggregation (PPDA) protocol, sensor data are partially exposed to neighboring trusted sensor nodes so that data aggregation can be achieved on the way to the sink node without revealing the actual data to the trusted sensor nodes or adversaries. There are many protocols available to achieve privacy-preserving data aggregation for WSNs. To the best of our knowledge, there is no comprehensive survey of the state of the art for privacy-preserving data aggregation for WSNs. The main challenge is to provide a new classification model for the PPDA protocols. First, the PPDA protocols can be classified based on a basis network topology, like routing protocols in the field of networking. In this type of classification, protocols are grouped into three classes; cluster, tree-structure and hybrid. But, PPDA protocols cannot be classified in this way because most PPDA protocols generally construct a common tree-structure as aggregation tree before transmitting data to the sink node. Secondly, a model can be used to classify protocols based on data collection methods. In this model, we can classify protocols into two groups: central collection and distributed collection. For example, query processing methods in spatial network databases can be grouped in this way. However, this classification model is not suitable for classifying the PPDA protocols because sensor nodes are resource-constraint in terms of processing speed, memory size and power supply. As a result, for distributed collection, a sensor node cannot store a large volume of sensor data for a long time. In a final classification model, we can classify the PPDA protocols based on a technique used to achieve privacy-preserving data aggregation for a WSN. The final classification model is more appropriate to classify PPDA protocols for WSNs because of the following three reasons. First, this model provides a broad range of groups available to classify protocols. The groups include perturbation, shuffling, hybrid and privacy homomorphism. Next, this model is appropriate to classify PPDA protocols for WSNs because the techniques used to achieve privacy-preserving data aggregation have their own peculiar features. For example, the privacy homomorphism technique is fast in terms of computation because it can execute arithmetic operations on cipher-text without decryption. As a result, the peculiar feature of a protocol can affect the issues of privacy-preserving data aggregation. At last, when the techniques are compared with each other in terms of privacy preserving and energy efficiency, this model leads us to explore which technique is the most suitable one in overall to achieve privacy-preserving data aggregation for WSNs.

**Figure 1.**
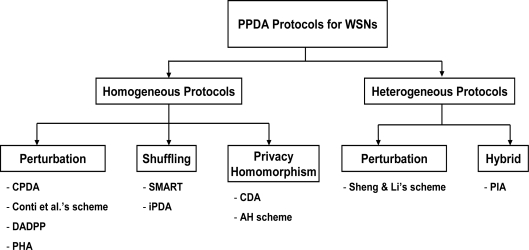
Classification of the existing PPDA protocols for WSNs.

Figure 1 illustrates the classification of the existing PPDA protocols for WSNs. The PPDA protocols are broadly categorized into two categories: homogeneous protocols and heterogeneous protocols. They are categorized based on the type of nodes in the WSNs, particularly the type of data aggregating nodes (aggregators). The aggregators can either be special (more powerful) nodes or regular sensor nodes. Moreover, the protocols are further divided into four groups; perturbation, shuffling, privacy homomorphism and Hybrid. As we mentioned above, this division is based on the techniques used to achieve privacy-preserving data aggregation for WSNs.

### 4.2. Homogenous protocols

In this type of protocols, all sensor nodes are homogeneous in a sense that they are same in terms of resources. The aggregators are normal sensor nodes which can sense data, perform aggregation and forward the aggregated value towards the sink node. Because all sensor nodes can play the role of the aggregators, this type of protocols causes no overhead when determining the positions of the aggregators. The protocols are of three kinds; perturbation, shuffling and privacy homomorphism.

#### 4.2.1. Perturbation

Perturbation technique is also known as data customization. In this technique, every sensor node uses encryption key and/or private or public seeds generated by randomization techniques [25,26], in order to hide the sampled data before transmitting them to a parent node. The perturbation-based protocols include CPDA, Conti *et al.*’s scheme, DADPP and PHA.

**Figure 2.**
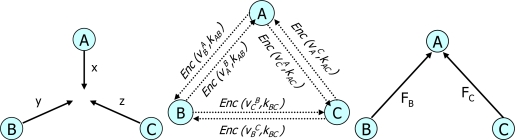
Message exchange within a cluster in the CPDA for (a) public seed broadcasting (b) customized data encryption & sending (c) assembled information broadcasting

**CPDA:** He *et al.* proposed the Cluster-based Private Data Aggregation (CPDA) [17] to achieve privacy-preserving data aggregation for WSNs. In the CPDA, sensor nodes are randomly grouped into clusters for creating an aggregation tree. Each cluster leverages the additive property of polynomials to calculate the desired aggregate value. At the same time, it guarantees that no individual node can know the data values of other nodes. The intermediate aggregate values in each cluster will be further aggregated on the way to the data sink along the aggregation tree. First, every sensor node in each cluster customizes its private data into polynomial form of order *k* – 1, where *k* is the total number of nodes in a cluster using shared (non-private) seeds and random numbers (private). Secondly, each sensor node encrypts its customized value by using a unique shared key between a sensor node and the other sensor nodes of the cluster. Thirdly, all nodes from the same cluster exchange their encrypted customized data with each other. Each sensor node has to encrypt and decrypt *O(N_c_)* messages, where *N_c_* is the number of sensor nodes in a cluster. Each node assembles all the data including its own by using the additive property of polynomials and sends them to their respective cluster leaders. After that, the cluster leaders deduce the aggregate value by computing the inverse of an *M × M* matrix where *M* is the number of cluster nodes. Finally, each cluster leader routes the derived sum of the cluster back towards the query server through the TAG routing tree [10]. Figure 2 shows message exchange within a cluster for the CPDA where there is a cluster leader *A* and two sensor nodes *B* and *C*.

However, since sensor nodes are resource-constrained, particularly in terms of the limited power and processing speed and the CPDA suffers from high communication and computation overheads. In addition, the CPDA can only tolerate the collusion to a certain threshold *i.e.*, the number of sensor nodes in a cluster minus two. If the number of colluding sensor nodes exceeds the threshold, the sensor nodes may collaboratively reveal the private information of some of the others. Although the threshold can be increased by expanding the size of the clusters, this will further increase communication overhead.

**CONTI *et al*.’s scheme:** The privacy-preserving data aggregation scheme by Conti *et al.* [19] first establishes twin keys for different pairs of sensor nodes in a network. Twin key establishment is an anonymous process that prevents each node in a pair from deriving the identity of the other node with which it is sharing a twin key. Then, for each aggregation phase, it uses an anonymous liveness announcement protocol to declare the liveness of each twin key. In the end, during the aggregation phase, each node encrypts its own value by adding shadow values computed from the lively twin keys it holds. In this way, the contribution of the shadow values for each twin key will cancel out each other and the correct aggregated result is finally obtained.

**Figure 3.**
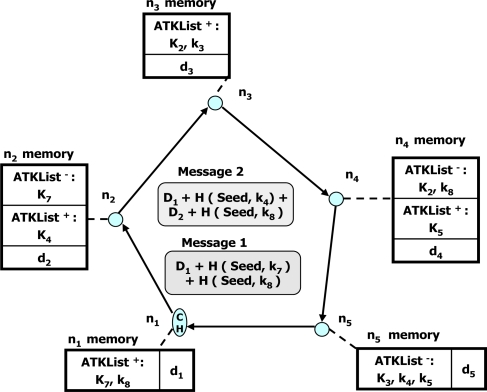
Data aggregation with shadow values.

**Figure 4.**
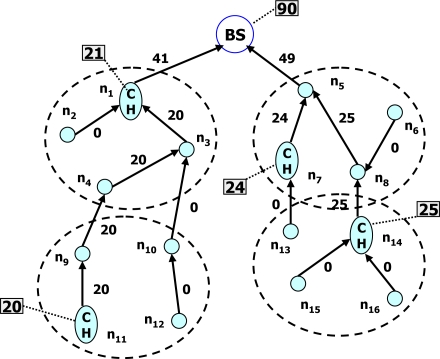
Aggregation of four-cluster aggregates.

There are three major steps on the scheme: *local cluster formation, twin-key establishment* and *data aggregation.* In the first step, nodes are grouped into several clusters and each cluster forms a different logical Hamiltonian circuit [27]. Each pair of neighboring nodes in the circuit shares a pair-wise key. In the twin-key establishment step, it is assumed that each node contains a pre-deployed ring of *K* symmetric key [28], randomly chosen from a larger common key pool of size *P*. Each node establishes a number of twin keys with the other nodes. In particular, a node *n_i_* establishes a twin key with another node (twin-node) in the cluster when *n_i_* is aware there is a node sharing a key with it. The twin keys are only known to the owners and they are established anonymously. In the data aggregation step, each cluster first computes the aggregated value of its nodes during which an aggregate is routed twice along the Hamiltonian circuit. Each node adds its own sensed value to the aggregate. For each live twin key, it adds (or removes) a corresponding shadow value. The cluster head obtains the correct aggregate for the cluster, because it is guaranteed that any shadow value added during aggregation by one node will be removed by another node that shares the same twin key. Then, by using a tree-aggregation hierarchical structure, the cluster head nodes contribute to the aggregate with the cluster aggregate. Finally, the base station (BS) receives the sum of the values owned by all of the cluster heads. Figures 3 and 4 illustrate data aggregation with shadow values and aggregation of the cluster aggregates of the proposed scheme, respectively. Although this scheme preserves the privacy of the data contributed by a sensor to the aggregate value, it has limited applications, since the information about the key pool is only known to the nodes manufacturer. In addition, this scheme incurs higher communication cost because not only an aggregate is routed twice along the Hamiltonian circuit, but also all nodes are involved while routing individual cluster aggregates to the BS. Furthermore, this scheme decreases the lifetime of the WSN, because all sensor nodes need longer idle times until the BS receives the aggregated value from the cluster heads.

**Figure 5.**
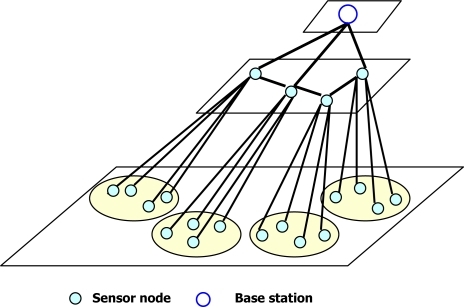
Hierarchical wireless sensor network.

**DADPP:** Data Aggregation Different Privacy-levels Protection (DADPP) [29] offers different levels of data aggregation privacy based on different node numbers for pre-treating the data. This protocol is inspired by the work of Shao *et al.* [30] in terms of different levels of privacy as well as the CPDA in terms of the privacy achieving method. In DADPP, a hierarchical wireless sensor network is first constructed in such that sensor nodes form several clusters each of which has a fixed cluster head below the energy efficient BS. According to the desired privacy level, all nodes within the same cluster are partitioned into multiple groups belonging to the same privacy level. Data are pretreated only in the same group and privacy levels are defined by the size of groups. The lowest privacy level consists of partitioned groups that have at least 3-sensor-nodes. The upper privacy level corresponds to portioned groups with 4-sensor-nodes. By analogy, if all sensor nodes of a cluster belong to a single group, they consider this case as the highest privacy level. The data aggregation process is similar to that of the CPDA. First, original data are pretreated in each group. Secondly, the cluster head aggregates all pretreated data. Finally, data are aggregated on the plane of the cluster head up to the BS. The hierarchical wireless sensor network is illustrated in Figure 5. Although DADPP reduces traffic by partitioning a cluster with *n* sensor nodes into multiple in-networks with pretreatment of groups according to the desired privacy-levels, it suffers from the inherent high communication and computation overheads. Furthermore, these overheads increase with increasing privacy level.

**PHA:** Zhang *et al.* [31] proposed the Perturbed Histogram-based Aggregation (PHA) to preserve privacy for queries targeted at special sensor data or sensor data distribution. The perturbation technique is applied to hide the actual individual readings and the actual aggregate results sent by sensor nodes. For this, every sensor node is preloaded with a unique secret number which is known exclusively by the sink and the node itself. Sensor nodes and the sink form a tree. The basic idea of PHA is to generalize the values of data transmitted in a WSN, such that although individual data content cannot be decrypted, the aggregator can still obtain an accurate estimate of the histogram of data distribution and thereby approximate the aggregates. In particular, before transmission, each sensor node first uses an integer range to replace the raw data. Next, with a certain granularity, the aggregator plots the histogram for data collected and then estimates aggregates such as *MIN*, *MAX*, *Median* and *Histogram*. Although the PHA supports many data aggregation functions, it has the following disadvantages. First, the final aggregated result is an approximation value of the sensor data rather than the real data. Secondly, the PHA requires a large size payload (message/data) because all sensor data need to be replaced by an integer range. Moreover, the bandwidth consumption of this protocol increases as the number of ranges increases. Finally, storing interval ranges to replace the original data consumes a significant amount of memory.

#### 4.2.2. Shuffling

In this technique, every sensor node slices its data into the fixed *J* number of data pieces and sends a data piece to the selected *J* – 1 number of neighboring sensor nodes. The remaining one piece of data is kept with it. After that, every sensor node assembles the received data pieces including its own piece of data and sends the assembled data to a parent node. SMART and iPDA are two shuffling-based protocols.

**Figure 6.**
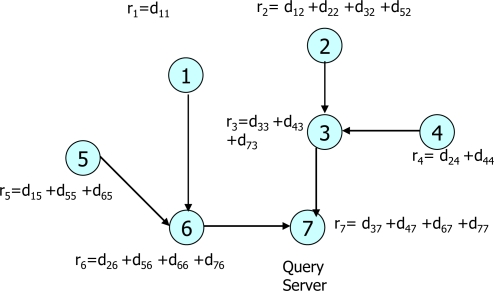
Data slices in SMART.

**SMART:** The Slice-Mix-AggRegaTe (SMART) by He *et al.* [17] achieves privacy-preserving data aggregation by hiding original data before the data transmissions. For this, each sensor node first customizes its private data by slicing it into a fixed number of pieces. Then, it sends data slices to a particular number of neighboring sensor nodes. After the data pieces are received from the neighboring sensor nodes, all sensor nodes calculate the aggregate value of the data slices so that the privacy of the sensor data can be preserved. In the SMART, each sensor node randomly selects a set of sensor nodes, say *J*, within *h* hops. When each sensor node slices its private data randomly into *J* pieces, *J* – 1 pieces are encrypted and sent to the randomly selected sensor nodes, keeping one data piece at the same sensor node. All the sensor nodes decrypt the data by using their shared keys and sum all the received slices. Each sensor node sends the sum to its parent. Finally, the root of the network is the ultimate aggregation point of all sensor data. Figure 6 shows an example of the SMART with seven nodes where *J* = 3 and hop *h* = 1. The notation *d_ij_* represents a slice of private data sent from node *i* to node *j* and *r_i_* represents the sum of sliced data. Although the SMART preserves the privacy of sensor data during their aggregation with low computation overhead, it generates a large number of messages in the network. In addition, like the CPDA, the SMART can tolerate only the collusion to a certain threshold, *i.e.*, the sum of out-degree and in-degree minus one.

**Figure 7.**
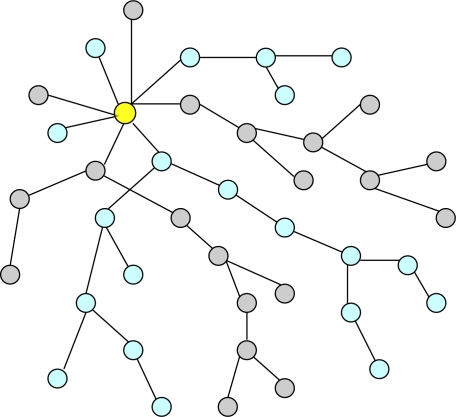
Two disjoint aggregation trees rooted at a base station.

**iPDA:** To address both privacy-preservation and integrity-protection for WSNs, He *et al.* [32] proposed a data aggregation protocol called integrity-Protecting Private Data Aggregation (iPDA). The iPDA resorts to a redundancy check by constructing two disjoint aggregation trees to achieve integrity. Each sensor node needs to send its reading to both aggregation trees and the inputs to both trees are equal. The disjoint aggregation trees perform data aggregation individually. Therefore, data pollution attacks can be detected at the base station by comparing aggregation results along the disjoint aggregation trees. If the aggregation results agree with each other, then the base station will accept the result. Otherwise, it will treat the data to have pollution attacks or node failures, or both and reject it. The iPDA achieves data privacy by using the slicing and assembling technique of the SMART. In this protocol, each participating sensor node first hides its individual data by slicing the data and sending encrypted data slices to different neighboring aggregators. Then, the aggregators collect and route aggregated results back to the base station. Figure 7 illustrates disjoint aggregation trees composed of sky-blue and grey nodes that are separately rooted at the base station (yellow node). However, the iPDA has a high communication overhead due to the slicing technique and each sensor node has to send its reading to both aggregation trees. In addition, it can tolerate the collusion of up to a certain threshold number of sensor nodes. Although the threshold can be raised by increasing the number of slices, it will further increase communication overhead. Moreover, the accuracy of the aggregated result is decreased because of the high data collision rate due to the large number of messages in the network.

#### 4.2.3. Privacy Homomorphism

Privacy Homomorphism (PH) has a special feature that allows arithmetic operations to be performed on cipher-text without decryption. This technique is fast and resource-efficient for privacy-preserving data aggregation, but it has a limitation that it performs only addition and multiplication operations. Before sensor data are sent to the aggregators, they are encrypted by using the respective keys of sensor nodes and they are added or multiplied without decryption. The CDA and AH scheme belong to PH-based protocols.

**Figure 8.**
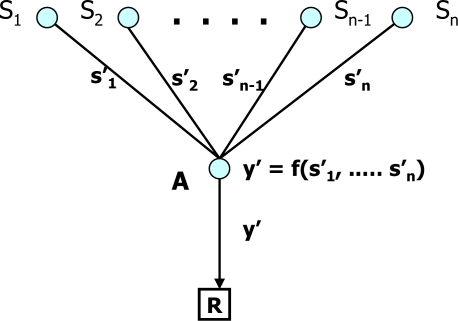
Data aggregation in CDA.

**CDA:** Girao *et al.* [33] proposed Concealed Data Aggregation (CDA) which conceals the process of data aggregation in WSN by using Domingo-Ferrer’s (DF) approach [34]. In this protocol, each sensor node splits its data into *d* parts *(d* ≥ 2*)*, encrypts them by using a public key and transmits them to the aggregator node. The aggregator node operates on the encrypted data, computes an aggregated value from the data without decryption and sends it to the sink. Next, the sink decrypts the encrypted aggregated value by using a private key to derive accumulated data. This allows the protocol to guarantee the privacy of the sensor nodes against a passive eavesdropper. Figure 8 illustrates data aggregation in the CDA where *S*_1_ to *S_n_*, *s*_1_ to *s_n_*, *A*, *y’* and *R* are sensor nodes, their respective encrypted data, aggregator node, encrypted aggregated value and sink node, respectively. Although the CDA is cheap in terms of computational resources, the CDA does not guarantee the privacy of individually sensed data against other nodes because the entire sensor nodes share the same encryption key with the BS. In addition, it does not protect against replay attacks and malicious aggregation where the contents of encrypted packets can be added as a summand to the aggregation result.

**AH scheme:** The Additively Homomorphic (AH) encryption technique by Castelluccia *et al.* [35] protects privacy against other sensor nodes. The main idea of this approach is to replace the *xor* (Exclusive-OR) operation typically found in stream ciphers with modular addition *(+)*. In the AH scheme, the encryption, decryption and aggregation processes are as given below where *c*, *m*, *k* and *M* are a cipher-text, a sensor data (message), a key shared with the BS and large integer, respectively.

$$\begin{array}{l} \mathrm{Encryption}:\;c=(m+k)\;\mathrm{mod}\;M\\ \mathrm{Decryption}:\;m=(c-k)\;\mathrm{mod}\;M\\ \mathrm{Aggregation}:\;c_{12}=\left(c_{1}+c_{2}\right)\;\mathrm{mod}\;M; \end{array}$$

The AH scheme assumes that every node *n_i_* shares a key *k_i_* with the BS. Basically, a node *n_i_* adds a random number to its sensed value where the random number is determined by the key *k_i_*. After receiving the encrypted aggregate, the BS filters out the correct aggregate by subtracting all the random numbers added by the nodes.

There are several disadvantages of the AH scheme, despite providing data privacy cheaply in terms of computational cost. First, the AH scheme is not scalable because the base station must know the keys of all aggregated packets in order to decrypt the received result. This requires additional communication costs for the transmission of all participated nodes’ *IDs* and corresponding security measures. There is no reliable solution to this problem, except that all nodes’ *IDs* are transmitted to the sink node as plaintext. None of the known solutions can provide both data integrity and confidentiality on the high level of security. Second, the AH scheme is vulnerable to malicious modifications of data by adding natural numbers to the cipher text. Finally, the AH scheme does not support data integrity.

### 4.3. Heterogeneous protocols

Because a heterogeneous network consists of more than one type of sensor nodes, a protocol running on more than one type of sensor nodes in a network is called heterogeneous protocol. The heterogeneous protocols consider an aggregator as a special node, instead of a regular sensor node. That is, the aggregator is not commonly used for data sensing, but for aggregating, storing and forwarding the aggregated value to the sink node. In heterogeneous protocols, the locations of the aggregators should be carefully determined because they are permanently fixed. The heterogeneous protocols are of two types; perturbation and hybrid.

#### 4.3.1. Perturbation

The perturbation technique has the same meaning as what we defined in the homogeneous protocols. The work proposed by Sheng *et al.* [36] is only the existing perturbation-based protocol.

**Figure 9.**
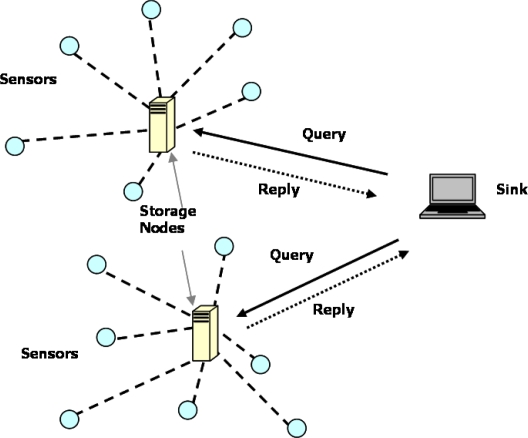
Two-tiered network model in Sheng and Li’s scheme.

**Sheng and Li’s scheme:** Sheng *et al.* [36] proposed a privacy-preserving storage scheme which associates a tag with each encrypted data in order to process a range query, by adopting the concept of bucketing [37] and indexing [38]. Their two-tiered network model consists of a powerful sink, regular sensor nodes, and storage nodes equipped with a large storage capacity, as shown in Figure 9.

In this scheme, every sensor generates environmental data values at a fixed rate and periodically submits the collected data in the form of coarse information to the closest storage node. The storage nodes first bucketize the mixture of original data into a few bins before storing them. Both the sensor node and the sink node have agreed on the same bucket partition. Before transmitting the data to the storage nodes, the data source first encrypts the data by using the key shared with the sink and then attaches a tag to the encrypted data indicating the bucket into which the data falls. When the sink node needs to process a range query over the data stored in the storage nodes, it obtains an approximate result based on the tags corresponding to the query range, instead of having storage nodes decrypting every data point and returning them. Then, through decryption at the base station, the real data set can be derived. However, the main problem of this scheme is the transmission delay, *i.e.*, sending data from sensors to the sink, which is critical for many tracking and event detection applications. In addition, this scheme does not consider the scenario of malicious modifications of sensor data.

#### 4.3.2. Hybrid

When a protocol uses more than one technique to achieve privacy-preserving data aggregation for WSNs, it belongs to the hybrid sub-group. PIA is only the hybrid-based protocol in this literature.

**Figure 10.**
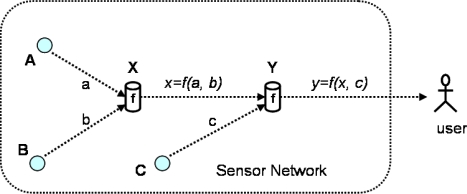
Data aggregation from sensors A, B and C with two aggregators X and Y.

**PIA:** To address Privacy-preserving Integrity-assured data Aggregation (PIA) for WSNs, recently, Taban *et al.* [39] proposed four distinct symmetric-key solutions. In their single aggregator model, an aggregator node is used as an intermediary between the user (*i.e.*, a third party) and the sensor nodes that aggregates the sensor data and forwards the query response to the user, as shown in Figure 10. The problem is that the user wants to verify the integrity of the received aggregate value whereas the network owner does not want the user to access the original data. The proposed four solutions to the problem are as follows. The first scheme uses homomorphic encryption to hide the data. In this scheme, homomorphism and Message Authentication Code (MAC) [40] are combined to construct an authenticated encryption scheme for the aggregator model. However, it is limited to additive homomorphism and only supports aggregation functions such as add, average and standard deviation. The second scheme adopts an Order Preserving Encryption Scheme (OPES) [41] to preserve the privacy of the distribution of the data. For example, OPES preserves the order of the data such that any pair of plaintexts *y*_1_ and *y*_2_, where *y*_1_ < *y*_2_, encrypt to ciphertexts *c*_1_ and *c*_2_, where *c*_1_ < *c*_2_. However, this framework is useful for any aggregation function which can be approximated by uniform sampling [24,42] and which relies only on the comparison operation. The third scheme directly uses the Secure Hierarchical In-networking Aggregation (SHIA) scheme [22] for adapting distributed integrity verification to the single aggregated model. Because the sensor nodes have access to all of the raw data, this scheme can support any aggregation function. However, the scheme has the communication overhead of *O(N)* messages per sensor node, where *N* is the total number of sensor nodes in a network. To achieve data privacy and integrity assurance the third scheme is improved by introducing a logical aggregation tree within the aggregator node. This is the fourth scheme know the *improved scheme* and each sensor node has the communication overhead of *O(log N)*. However, the scheme only supports decomposable functions, such as *Mean, Standard Deviation, Count* and *Min/Max.*

## 5. Comparative Study of PPDA Protocols

In this section, we present a comparison result of the existing PPDA protocols for WSNs. The comparison result is based on the following metrics.

- *Communication cost (CMC):* This is the number of messages generated in the given WSN. To evaluate CMC, we use *High, Medium and Low*. The communication costs of protocols belong to *High, Medium and Low* when the numbers of message generated (m) per sensor node is *m* ≥ 3, 3 > *m* > 1, *m* = 1, respectively. For example, iPDA, CPDA and CDA protocols belong to *High, Medium and Low* because they generates 5, 2 and 1 message(s) per sensor node, respectively.
- *Computation cost (CPC):* This is a processing overhead to achieve privacy-preserving data aggregation. The CPC can be divided into three types; *High, Medium and Low*. A protocol has a high cost if its sensor node performs many encryption/decryption, arithmetic operations and other computation like matrix operations. A protocol has a medium cost if its sensor node performs a couple of encryptions/ decryptions and some arithmetic operations. If a sensor node performs a few arithmetic operations and just encrypts its data, a protocol has a low cost. For example, because a sensor node in the AH scheme just encrypt data, the AH scheme has a low cost. The CPC of the PHA is medium because its sensor node performs two encryption and a few addition operations. Lastly, the CPC of the DADDP is high because its sensor node performs about four pairs of encryption/decryption and many addition/multiplication operations.
- *Data pollution (DP):* This is a logical operator for detecting the malicious modification of sensor data. If the malicious modification is detected, the metric gives *Yes*, otherwise it gives *No*. because all of the current PPDA protocols do not support DP feature, they are labeled *No*.
- *Privacy support type (PST):* There are two types of privacy support. The first one is used to preserve the privacy of raw sensor data from other trusted sensor nodes in the same network whereas the second one is used to preserve their privacy from adversaries/outsiders. *Both* and *Outside*r are two labeling for this metric. If a protocol supports data privacy against the trusted sensor nodes and outsiders, it is labeled *Both*. The CPDA belongs to *Both*. On the other hand, when the privacy of data is protected only from adversaries not being in the same network, it is labeled *Outsider*. The Sheng & Li’s scheme belongs to *Outsider*.
- *Delay (DLY):* This is the time lag which takes for the aggregated data to reach the sink from the source nodes, due to the overhead of protocol execution. To evaluate DLY, we use *High, Medium and Low*. For example, Conti *et al.*’s scheme has high transmission delay because its data rotate two rounds in the Hamiltonian circuit of cluster members before being sent to the aggregation tree. The SMART has medium transmission delay because its data are encrypted/decrypted before they are aggregated. The transmission delay of CDA is low because its data are aggregated in cipher texts form.
- *Memory consumption (MC):* This is the amount of main memory space required for storing keys, variables and integer ranges in a sensor node. The MC has three values; *High*, *Medium* and *Low*. For example, the PHA requires high memory consumption because it needs about 1 Kbytes to store 200 keys, random numbers and data mapping ranges. The SMART requires medium memory consumption because it needs a few hundreds of bytes to store many keys and some variables. Lastly, the memory consumption of CDA is low because only about 10 bytes are needed to store some keys and variables.
- *Data integrity (DI):* This is a metric used to check whether a protocol supports data integrity or not. If a protocol supports this feature (the DI value is *Yes*), we can assure that sensor data have been correctly aggregated. Otherwise, the DI value is *No*. For example, because the iPDA supports data integrity, its DI value is *Yes*. The DI value of SMART is *No* because it does not support this feature.
- *Data loss resiliency (DLR):* This is a behavior to cope with a message dropping condition in WSNs so that the aggregated result obtained can be real. The DLR value has *Yes* or *No*. For example, because the Conti *et al.*’s scheme supports the DLR property but the AH scheme does not, they are labeled *Yes* and *No*, respectively.
- *Accuracy (ACU):* This is the deflection of the aggregated value obtained from the real value of sensors data. To evaluate ACU, we use *High, Medium and Low*. For example, the CPDA has the high accuracy of the aggregated data because its computation is based on exact real numbers. The PIA has medium accuracy because its computation depends on both the exact numbers and random numbers. Lastly, the PHA has low accuracy because its final aggregated result is derived from data mapping range.
- *Aggregation function (AF):* This deals with how many aggregation functions a protocol can support among *Sum, Average, Count, Standard Deviation, Variance, Min, Max, Median and Histogram.* There are two groups for AF; *Numerous* and *Few*. For example, the PHA supports numerous aggregation functions, like *Sum, Average, Count, Min, Max, Median, Histogram,* whereas the CDA supports only a few functions, like *Sum, Count* and *Average*.
- *Payload size (PLS):* This is the real information size after encryption/keying, perturbation or mapping, in order to preserve the privacy of actual data. The payload size is not the whole packet size and can be divided into *Large, Medium and Small*. For example, the payload size of the SMART is small (a few bytes) because the sample data are encrypted by using a key. The payload size of the AH scheme is medium (more than 10 bytes) because the sample data is encrypted by a key and the IDs of contributing sensor nodes are appended to the payload. The PHA needs a large payload size because it should carry a large range of encrypted data obtained from data mapping.
- *Energy consumption (EC):* This is the overall amount of energy dissipated by WSNs to collect sample data from source nodes. To evaluate EC, we use *High, Medium and Low*. Because data communication is a dominant energy consuming process in WSNs, we evaluate the protocols based on the size of payloads and the number of messages generated in the networks. For example, the CDA requires low energy consumption because its payload size is small and each sensor node generates only one message. The Sheng and Li’s scheme requires medium energy consumption because its payload size is large, even though a sensor node generates a single message. Lastly, the energy consumption of the SMART is high because a sensor node generates at least three messages.

Table 1 shows a comparison of the PPDA protocols for WSNs. The comparison presents a lot of remarkable information about the current status of the PPDA protocols. First, Conti *et al.*’s scheme, DADPP, SMART and iPDA generate multiple messages in WSNs during execution, whereas the remaining protocols are cheap in terms of computation cost. The message transmission cost is always the dominant energy consumption factor in WSNs. Secondly, the computational overhead is one of the responsible factors of transmission delay and energy consumption. CPDA, Conti *et al.*’s scheme and DADPP are computationally expensive, whereas SMART, AH scheme and CDA have low computational overhead. The rest of the PPDA protocols are medium in terms of computation cost. Thirdly, the detection of malicious modifications of sensor data on their ways to the sink is crucial for achieving a true aggregation result, but unfortunately, none of the protocols supports this feature. Fourthly, since privacy is one of the main design principles of a PPDA protocol, all protocols need to be robust in order to preserving data privacy against adversaries. However, the CDA, AH scheme, Sheng and Li’s scheme and PIA are vulnerable to leaking the privacy of raw sensor data during transmission from one sensor node to others of the same WSN. Fifthly, a data propagation delay is critical to many applications, such as fire detection, so this issue should be addressed by a PPDA protocol. There are many protocols designed with a tolerable delay time, except Conti *et al.*’s scheme, Sheng and Li’s scheme and iPDA. Sixthly, due to the limited memory of a sensor node, the space occupied in main memory by the variables, keys and integer ranges is always a matter of concern in WSNs. But, there are only two protocols, the CDA and AH scheme, which are very efficient in terms of memory usage. Seventhly, because data integrity assures the correctness of the aggregated data and critical decisions are made based on the aggregated result, it is necessary to support data integrity features by using a PPDA protocol. However, only a few protocols, such as iPDA, Sheng and Li’s scheme and PIA support data integrity. Next, lost data must be considered during data aggregation; otherwise the final aggregated value does not provide an exact picture of a WSN. There are many protocols, including CPDA, Conti *et al.*’s scheme, DADPP, PHA, CDA, Sheng & Li’s scheme and PIA, supporting data loss resiliency. Then, the accuracy of the final aggregated result is always desirable for the relevancy of the system. Many of the existing PPDA protocols provide a high degree of accuracy, such as CPDA, Conti *et al.*’s scheme, DADPP, Sheng and Li’s scheme, AH scheme and CDA. After that, because concurrent applications running over the same WSN may require the computation results of different aggregation functions, it is mandatory for a PPDA protocol to support many aggregation functions, such as *Sum, Min* and *Max*. But, only PHA and PIA can support multiple aggregation functions whereas most of the existing protocols can support only a few aggregation functions. Furthermore, from the communication point of view, a small size message is expected in WSNs. Except from Sheng and Li’s scheme and PHA, the size of the message in rest of the PPDA protocols is not large. In the end, because of limited power supply, the lifetime of a WSN directly depends on the available energy of sensor nodes. Protocols which need more energy to collect sampled data from WSNs always provide short-time services, compared with those protocols which need less energy for the same purpose. Therefore, a protocol with less energy consumption is considered efficient in WSNs. However, there are still several protocols which can not properly address the energy issue of WSNs, such as Conti *et al.*’s scheme, DADPP, SMART and iPDA. Both CPDA and PHA need moderate amount energy. Although CDA, AH scheme, B, Sheng *et al.* and PIA require a low amount of energy they preserve data privacy only against outsiders. This implies that protocols preserving data privacy of one sensor node against others of the same WSN generate multiple messages. As a result, they consume a significant amount of energy.

In summary, although all the aforementioned metrics are important to evaluate the efficiency of a PPDA protocol, we argue that Privacy Support Type (PST), Accuracy (ACU), Aggregation Function (AF) and Energy Consumption (EC) are the four most relevant parameters to be considered. On the basis of the four metrics, we believe that CPDA and PHA are the most appropriate two protocols of all the existing PPDA protocols to achieve private data aggregation. The reasons are as follows. First, both CPDA and PHA can preserve the privacy of a sensor data from the others of the same WSN as well as from adversaries. Secondly, the CPDA achieves high accuracy of the aggregated result, although it needs relatively a high amount of energy, whereas PHA achieves enhanced accuracy by increasing the interval ranges at the expense of an additional amount of energy. Finally, the PHA can support many aggregation functions and the CPDA can also support the most basic aggregation functions, such as *Sum* and *Average*.

The technique used to achieve privacy-preserving data aggregation for WSNs is the core of our classification model for PPDA protocols. So, based on the Table 1, we describe some important remarks on perturbation, shuffling, privacy homomorphism and hybrid techniques. First, the perturbation technique of homogeneous protocols (PT1) and shuffling technique can protect data privacy from both neighboring sensor nodes and outsiders. But, these two techniques consume a significant amount of energy due to many data transmissions and large computational overhead. On the other hand, the perturbation technique of heterogeneous protocols (PT2), privacy homomorphism and hybrid technique preserve data privacy only from outsiders. Therefore, they are energy efficient because they generate the less number of data traffics in the network. Secondly, PT1, PT2 and hybrid technique provide the data-loss-resiliency feature whereas shuffling technique does not provide the data-loss-resiliency. On the other hand, privacy homomorphism depends on each protocol for offering the data-loss-resiliency. Thirdly, the privacy homomorphism requires less main memory space than PT1, PT2, shuffling, hybrid technique. Specially, PT2 requires a large amount of main memory space. Fourthly, both PT1 and PT2 techniques have larger payload size than those of shuffling and hybrid techniques. Fifthly, only the privacy homomorphism has low transmission delay whereas the rest of techniques have medium or high delay. Sixthly, both PT2 and hybrid technique supports data integrity whereas PT1 and privacy homomorphism does not support it. On the other hand, the shuffling technique is specific to each protocol to support data integrity. Seventhly, the hybrid technique provides many aggregation functions whereas PT1, PT2, shuffling and privacy homomorphism provide the most basic aggregation functions. At last, shuffling, privacy homomorphism and hybrid technique use smaller messages in size than PT1 and PT2. Conclusively, we can see from the viewpoint of both privacy preservation and energy consumption that the perturbation technique of the homogeneous protocols is the best one.

## 6. Future Directions

Although the present research addresses many of the issues on privacy-preserving data aggregation, there are still many research areas which must be studied. Here we list some important ones. First, the current trend of research on privacy-preserving data aggregation considers static networks. But many real life scenarios, such as sensing the health status of athletes and patients, require dynamic networks. Therefore, privacy-preserving data aggregation in dynamic environments is a possible future research direction. Second, many critical decisions are made on the basis of the aggregated results obtained. Therefore, the aggregated results should be free from malicious modifications of sensor data, *i.e.*, false data injections, by any compromised node (particularly by a compromised aggregator). Detecting false data injections in WSNs is a challenging task. Hence, the development of a privacy-preserving data aggregation scheme that avoids the problem of malicious modifications of sensor data on their way to the sink could be an interesting issue for future research. Third, since sensor data is highly correlated, data aggregation can be achieved by employing source coding techniques. Using the idea of source coding may seamlessly integrate data privacy and aggregation. For this reason, there is significant scope for future work on privacy-preserving data aggregation based on source coding. Fourth, data aggregation and integrity checking threaten privacy preservation of sensor data because for supporting comparative aggregation functions, especially *Min/Max* functions, data aggregators need to know the raw data of the sensor nodes for data integrity checking. In this way, individual sensors data are disclosed to other sensor nodes, thus resulting in the violation of privacy preservation. Therefore, it will be worthwhile to address both comparative aggregation functions and data integrity checking without needing the raw data of sensor nodes, as future research. Fifth, data aggregation saves the precious energy of WSNs by reducing the number of messages transmissions whereas a protocol for privacy preservation of sensor data needs a significant amount of energy. Therefore, future work needs to explore the combined impact of privacy preservation and data aggregation on the performance of WSNs and examine the tradeoff between data aggregation and privacy preservation. Last but not least, because WSNs are not reliable, it cannot be expected that all nodes will reply to all requests. Therefore there needs to be a reliable and scalable mechanism for communicating the *IDs* of the contributed nodes to the sink node. Since TinyOS-based sensor nodes have a limited payload size (29-bytes), support for the transmission of the *IDs* of contributed sensor nodes along with aggregated data to the sink node for large-scale WSNs is a possible future research direction for privacy-preserving data aggregation.

## 7. Concluding Remarks

Wireless sensor networks (WSNs), which are one of the attractive topics in academia, are penetrating the physical world through different application fields that are impacting our daily lives. Thus, there are many opportunities to develop real applications for WSNs. Data aggregation techniques increase the lifetime of sensor nodes providing continuous services to users because they reduce the number of message transmissions. On the other hand, many applications require the privacy of sensor data during their collection in the sink. In this paper, we first explored the design principles, issues and challenges for PPDA protocols. Then, we investigated different protocols to achieve privacy-preserving data aggregation in WSNs and classified them into *homogeneous* and *heterogeneous protocols* on the basis of the types of data aggregators. The former uses regular sensor nodes as data aggregators whereas the latter uses special nodes – different from regular sensor nodes in terms of available resources – as data aggregators. We further categorized these protocols into four groups on the basis of the techniques applied to achieve data privacy. They are *perturbation, shuffling, PH and hybrid*. After that, we presented a comprehensive comparison of the existing PPDA protocols in terms of many meaningful metrics, such as supporting type of privacy and energy consumption. Our comparison result provides an insight into the strengths and weaknesses of the existing PPDA protocols for WSNs. Finally, we provided some future research directions in the field of privacy-preserving data aggregation for WSNs. We believe that our comparison result along with future research directions will definitely encourage many researchers improving the existing protocols and designing new PPDA protocols for achieving privacy-preserving data aggregations in WSNs, efficiently.

## Figures and Tables

**Table 1.** Comparison result of PPDA protocols for WSNs.

**Metrics**			**CMC**	**CPC**	**DP**	**PST**	**DLY**	**MC**	**DI**	**DLR**	**ACU**	**AF**	**PLS**	**EC**
**Protocols**														
**Homogeneous Protocols**	Perturbation	*CPDA*	M	H	N	B	M	M	N	Y	H	F	M	M
		*Conti et al.’s scheme*	H	H	N	B	H	M	N	Y	H	F	M	H
		*DADPP*	H	H	N	B	M	M	N	Y	H	F	M	H
		*PHA*	M	M	N	B	L	H	N	Y	L	U	G	M
	Shuffling	*SMART*	H	L	N	B	M	M	N	N	H	F	S	H
		*iPDA*	H	M	N	B	H	M	Y	N	H	F	S	H
	Privacy Homomorphism	*CDA*	L	L	N	O	L	L	N	Y	H	F	S	L
		*AH scheme*	L	L	N	O	L	L	N	N	H	F	M	L
**Heterogeneous Protocols**	Perturbation	*Sheng & Li’s scheme*	L	M	N	O	H	H	Y	Y	H	-	G	M
	Hybrid	*PIA*	L	M	N	O	M	M	Y	Y	M	U	S	L

*Legend:* H = High; M = Medium; L = Low; N = No; Y = Yes; B = Both; O = Outsider; U = Numerous; F = Few; G = Large; S = Small; “-” = Not Mentioned
